# Discrepancies between self-reported current and ideal sleep behaviors of adolescent athletes

**DOI:** 10.5935/1984-0063.20190122

**Published:** 2020

**Authors:** Melissa L Anderson, Reid J. Reale

**Affiliations:** Gatorade Sports Science Institute, PepsiCo, Inc. - Bradenton - FL - United States

**Keywords:** Team Sports, Pittsburgh Sleep Quality Index, Adolescents

## Abstract

It is well documented that many adolescents are not meeting current sleep duration recommendations, with a growing body of literature suggesting adolescent athletes also fall into this category. What is less known is the relationship between current and ideal sleep behaviors. We sought to quantify sleep behaviors in a group of athletes and to understand how their current behaviors compare to their self-reported ideal behaviors. One hundred ninety six competitive, male and female athletes (15.7 ± 1.3 y) completed the Pittsburgh Sleep Quality Index (PSQI) and a questionnaire that captured usual sleep habits. The PSQI was analyzed for habitual bedtime, wake time, sleep duration, and sleep quality. The usual sleep habits questionnaire was analyzed for ideal bedtime, wake time, and calculated sleep duration. Reported mean sleep duration was 7:45 ± 1:06 h:min. Actual bedtime was later (+0:44 ± 0:05 h:min, *p<*0.001) than ideal bedtime, actual wake time was earlier (-0:50 ± 0:08 h:min, *p<*0.001) than ideal wake time, and actual sleep duration was less (-2:11 ± 1:27 h:min, *p<*0.001) than ideal sleep duration. Adolescent athletes are not meeting current sleep duration recommendations and there are significant discrepancies between self-reported current and ideal sleep behaviors in this group.

## INTRODUCTION

Leading health organizations such as the Centers for Disease Control and Prevention (CDC), as well as professional organizations such as the American Academy of Sleep Medicine (AASM) and the National Sleep Foundation (NSF) all recommend adolescents obtain 8-10 hours of sleep per night^1,2^. Since the introduction of sleep-duration tracking to the CDC’s large-scale Youth Risk Behavior Survey (YRBS) in 2007, it has been well documented that most adolescents (roughly two-thirds of the over 50,000 adolescents surveyed) obtain insufficient sleep (defined as seven or less hours of sleep) on the average school night^3,4^. A similar pattern of inadequate sleep has also been documented in adolescent athletes, with Milewski et al.^5^ reporting almost 80% of 112 athletes surveyed sleeping 7 hours or less per night.

This seemingly common sleep shortage should cause alarm because consistently obtaining a less-than-ideal sleep duration has been associated with lower cognitive performance^6^ and emotional wellness^7^, and unfavorable changes in health indices such as waist and hip circumferences and body fat^8^, while later bedtimes have been associated with poorer school performance^9^ in this population. For athletes specifically, less sleep has also been linked with an increase in risk for negative mood states^10^, injuries^5,11^, and decrements in sport-specific performance^12^, while obtaining more sleep, in the form of either nighttime sleep or a nap, can improve sport-specific performance^13-15^.

While there is a growing body of literature that would suggest why adolescents, and especially adolescent athletes, should sleep more, research has primarily focused around actual behaviors. To date, little to no research has focused on the role of the *desire* of the adolescent athlete to sleep more. Presumably, this relationship (or lack thereof), may be an important consideration for practitioners as they determine how to most successfully increase sleep duration in this population. Therefore, the purpose of this study was to quantify current self-reported sleep habits in a group of adolescent athletes and to understand how their current habits compare with their self-reported ideal sleep durations. Additionally, we sought to determine if there were any differences by sex or by sport.

## METHODS

### Participants

One hundred ninety six competitive, adolescent athletes (156 male, 40 female; 15.7 ± 1.3 y) from a mixture of individual and team-based sport disciplines (Table 1) participated in the study. All athletes were recruited from a predominantly live-in, sport-focused middle/high school and training academy. The student-athletes in this environment split their day between school and sport (approximately 4 hours of each) with the first scheduled activity typically starting between 07:30 and 08:00 and the last scheduled activity concluding between 17:00 and 17:30. This study was approved by the Sterling Institutional Review Board (Atlanta, GA; sterlingirb.com; an independent review board not affiliated with the authors’ institution) and has, therefore, been performed in accordance with the ethical standards laid down in the 1964 Declaration of Helsinki. Each participant and his/her parent/legal guardian were advised of the experimental procedures and associated risks before giving their written informed assent or consent, respectively.

**Table 1:** Subject characteristics by sport.

	All Athletes	Basketball	Soccer	Baseball	Football	Golf	Lacrosse	Tennis
n	196	23	50	32	21	22	20	28
Male / female	156/40	18/5	33/17	32/0	21/0	16/6	19/1	17/11
Age (y)	15.7 ± 1.3	15.8 ± 1.3	15.5 ± 1.4	16.2 ± 1.3	15.9 ± 1.4	15.8 ± 1.3	16.0 ± 1.0	15.3 ± 1.2
Daily training duration (min)	126 ± 67	123 ± 53	152 ± 63^a^	104 ± 75^b^	113 ± 37	95 ± 67^ac^	97 ± 38^d^	162 ± 78^bcd^

Values that share the same letter are significantly different from one another. Data shown as means ± SD.

### Procedures

Each athlete completed paper and pen versions of the Pittsburgh Sleep Quality Index (PSQI)^16^ and a questionnaire, which captured usual sleep habits during a single laboratory visit. The PSQI was analyzed to determine self-reported habitual bedtime, sleep onset latency, wake time, and sleep duration, as well as sleep quality as measured by the global PSQI score (0-21 scale with a score ≥ 5 indicative of poor sleep quality). The questionnaire which captured usual sleep habits was analyzed to determine self-reported ideal bedtime (“To feel my best, I should go to bed at?”), wake time (“To feel my best, I should get up at?”), and calculated sleep duration. Self-reported daily training duration (“I usually exercise at (insert time) for (insert) minutes”) was also obtained from the usual sleep habits questionnaire. For both surveys, if any answer was written as a range (e.g.: 07:30 - 08:00), the midpoint was used for the analysis.

### Data analysis

Distribution of data was assessed for normality using D’Agostino & Pearson normality tests. When data were not normally distributed, log transformations were performed to achieve normality where possible.

Comparisons between sexes and between ideal and current behaviors were conducted using unpaired T-Tests when data were normally distributed and by Mann-Whitney U-tests when data were not normally distributed. Comparisons between sports were conducted using analysis of variances (ANOVA) with Tukey-post hoc tests when data were normally distributed and by Kruskal-Wallis tests followed by Dunn’s multiple comparisons test when data were not normally distributed.

Pearson correlation coefficient (r) as well as coefficient of determination (r^2^) values were calculated to assess the association between age and: bedtime, wake time, sleep duration, sleep onset latency, global PSQI score, ideal bedtime, ideal wake time, ideal sleep duration, and ideal sleep duration minus actual sleep duration. Pearson correlation coefficient (r) and coefficient of determination (r^2^) values were also used to assess potential associations between sleep duration and: bedtime and wake time, as well as for training duration and: sleep onset latency, bedtime, wake time, global PSQI score, sleep duration, ideal bedtime, ideal wake time, ideal sleep duration, and ideal sleep minus actual sleep duration.

When data were transformed to achieve normality, statistical analyses were completed on the transformed data, with back transformed data being displayed in tables and figures for ease of visualization^17^. In recognition of the multiple comparisons made within the dataset, significance was set at *p*=0.0125. Data are displayed as mean ± standard deviation unless otherwise stated. All statistical analysis was performed using GraphPad Prism version 7.04 for Windows, (GraphPad Software, La Jolla California USA).

## RESULTS

### Subject characteristics

Table 1 displays subject characteristics by sport. In total 196 athletes were included in the data set, including 156 males and 40 females. Sport had no main effect on age (*p*=0.2620), however did have a main effect on daily training duration (*p*=0.0004). Specifically, post hoc-tests suggested soccer athletes reported significantly greater daily training duration than golf athletes did (*p*=0.0091), and baseball, golf and lacrosse athletes reported significantly lower daily training duration than tennis athletes (*p*=0.0089, *p*=0.0052, *p*=0.0088, respectively).

### Sleep habits by sport

**Figure 1:**
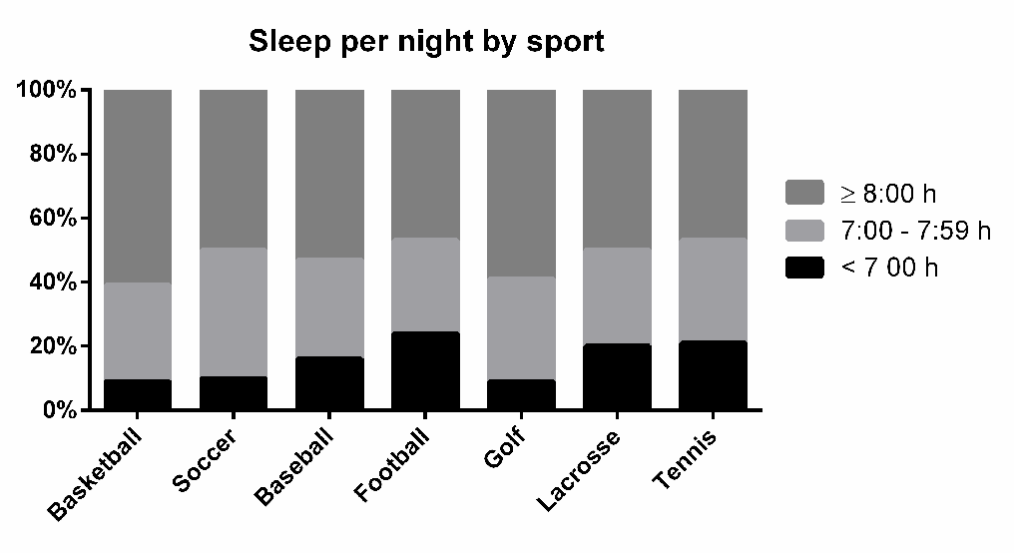
Categorization of sleep duration by sport.

Table 2 displays sleep habits by sport. The overall proportion of athletes with sleep duration of ≤ 6:59, 7:00-7:59, and ≥ 8:00 h:min was 14.8%, 33.2%, and 52.0%, respectively, with results by sport shown in Figure 1. ANOVAs indicated no main effects of sport on bedtime (*p*=0.0930), sleep per night (*p*=0.7363), or sleep onset latency (*p*=0.6594). Kruskal Wallis test showed a main effect of sport on wake time (*p*<0.0001). Dunn’s post hoc tests suggested significant differences in wake time between baseball and tennis (*p*=0.0012) and golf and tennis (*p*=0.0086), with differences between football and tennis (*p*=0.0307), and lacrosse and tennis (*p*=0.0326) approaching significance.

**Table 2:** Current self-reported sleep habits by sport.

	All Athletes	Basketball	Soccer	Baseball	Football	Golf	Lacrosse	Tennis
Bedtime	22:39±0:47	22:36±0:49	22:31±0:41	22:45±0:40	22:48±0:48	22:41±0:44	23:00±1:03	22:23±0:47
Wake time	07:01±1:07	7:27±1:04	6:35±0:32	7:23±1:17^a^	7:09±1:04	7:13±1:01^b^	7:32±1:48	6:24±0:45^ab^
Sleep onset latency (min)	15±11	18±17.5	13±7.0	17±10.3	16±10.4	13±7.7	18±16.4	15±10.3
≤ 15 min	50.5%	52.2%	52.0%	40.6%	52.4%	63.6%	50.0%	46.4%
16 - 30 min	34.7%	34.8%	40.0%	37.5%	19.0%	31.8%	25.0%	42.9%
31 -60 min	13.8%	8.7%	8.0%	21.9%	28.6%	4.5%	20.0%	10.7%
> 60 min	1.0%	4.3%	0.0%	0.0%	0.0%	0.0%	5.0%	0.0%
Sleep duration (h:min)	7:45±1:06	7:48±1:04	7:41±0:47	7:43±1:02	7:39±1:21	8:08±1:10	7:57±1:33	7:30±1:06

Values that share the same letter are significantly different from one another. Data shown as means ± SD.

### Ideal sleep habits by sport compared to current sleep habits

Table 3 displays ideal sleep habits by sport. Results of T-tests comparing all sports combined ideal and actual habits revealed actual bedtime was significantly later (+0:44 ± 0:05 h:min, *p*<0.001) than ideal bedtime, actual wake time was significantly earlier (-0:50 ± 0:08 h:min, *p*<0.001) than ideal wake time, and actual sleep duration was significantly less (-2:11 ± 1:27 h:min, *p*<0.001) than ideal sleep duration. ANOVAs suggested no main effects of sport on ideal bedtime (*p*=0.037), ideal wake time (*p*=0.065), ideal sleep duration (*p*=0.369), or ideal sleep minus actual sleep duration (*p*=0.6088).

**Table 3:** Self-reported ideal sleep habits by sport.

	All Athletes	Basketball	Soccer	Baseball	Football	Golf	Lacrosse	Tennis
Ideal bedtime	21:55 ± 0:49	21:40 ± 1:03	22:02 ± 0:36	22:10 ± 0:55	21:46 ± 0:42	21:43 ± 0:52	22:14 ± 1:02	21:39 ± 0:30
Ideal wake time	07:50 ± 1:17	7:55 ± 1:13	7:40 ± 1:11	8:26 ± 1:29	8:01 ± 1:18	7:27 ± 1:20	8:03 ± 1:37	7:26 ± 1:09
Ideal sleep duration (h:min)	9:55 ± 1:25	10:15 ± 1:34	9:38 ± 1:04	10:27 ± 1:27	10:15 ± 1:32	9:42 ± 1:33	9:49 ± 1:40	9:47 ± 1:23
Ideal sleep minus actual sleep duration (h:min)	-2:11 ± 1:27	-2:24 ± 1:47	-1:58 ± 1:18	-2:34 ± 1:21	-2:43 ± 1:19	-1:31 ± 1:19	-1:46 ± 1:16	-2:17 ± 1:38

Data shown as means ± SD.

### Sleep habits by sex

Following un-paired T-Tests and Mann Whitney-U Tests (as appropriate), no differences between sexes were found for age (male 15.8 ± 1.3 y vs female 15.5 ± 1.4 y, *p*=0.2395), bedtime (male 22:41 ± 0:41 vs female 22:29 ± 0:53, *p*=0.0604), wake time (male 07:04 ± 1:05 vs female 06:48 ± 1:15, *p*=0.0468), sleep duration per night (male 7:45 ± 1:07 h:min vs female 7:46 ± 1:04 h:min, *p*=0.9943), sleep onset latency (male 0:15 ± 0:10 h:min vs female 0:16 ± 15 h:min, *p*=0.7337), global PSQI score (male 4.0 ± 2.2 vs female 4.4 ± 2.5, *p*=0.4939), ideal bedtime (male 21:53 ± 0:43 vs female 21:59 ± 1:06, *p*=0.4563), ideal wake time (male 07:43 ± 1:17, female 08:21 ± 1:27, *p*=0.019), ideal sleep duration (male 9:49 ± 1:22 h:min vs female 10:22 ± 1:33 h:min, *p*=0.0411), or ideal sleep minus actual sleep duration (male -2:07 ± 1:50 h:min vs female -2:32 ± 1:53 h:min, *p*=0.2330).

### Correlations

Table 4 displays correlations between selected variables of interest. Significant correlations were found between age and: bedtime, sleep duration, global PSQI score, ideal bedtime, and ideal sleep minus actual sleep duration; as well as sleep duration and: bedtime and wake time. Additionally, training duration was positively correlated with ideal sleep duration. However, training duration was not correlated with any other current or ideal sleep variables.

**Table 4:** Correlations between selected variables of interest.

Association tested	Pearson r value	*p* =	95% CI	r^2^
Age and bedtime	0.358	<0.0001*	0.229:0.474	0.128
Age and sleep duration	-0.296	<0.0001*	-0.4192:-0.1631	0.087
Age and global PSQI score	0.204	0.0040*	0.066:0.335	0.041
Age and ideal bedtime	0.227	0.0021*	0.084:0.361	0.052
Age and ideal sleep duration	-0.023	0.7568	-0.169:0.123	<0.001
Age and ideal sleep minus actual sleep duration	-0.191	0.0100*	-0.329:-0.046	0.037
Sleep duration and bedtime	-0.427	<0.0001*	-0.535:-0.305	0.183
Sleep duration and wake time	0.488	<0.0001*	0.374:0.588	0.238
Sleep duration and ideal sleep duration	-0.1105	0.1398	-0.2527:0.03640	0.012
Training duration and ideal sleep duration	0.2225	0.0032*	0.0763:0.3594	0.050

### Global PSQI scores

**Figure 2:**
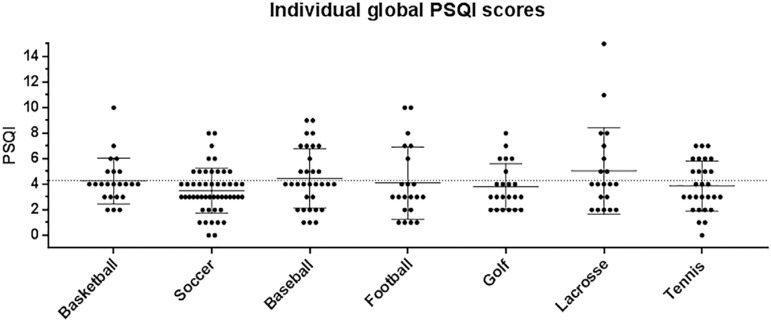
Individual global PSQI scores by sport.

Figure 2 displays Tukey box and whisker plots of global PSQI score by sport. The combined mean global PSQI score for all sports was 4.1 ± 2.3. ANOVA revealed no main effect for sport on global PSQI score (*p*=0.029).

## DISCUSSION

The main finding from the present study is that close to half of all adolescent athletes, both male and female, and across all sport types included are not meeting the minimum sleep recommendation of 8-10 hours of sleep per night. Mean sleep quality was acceptable in our study; however, there was large interpersonal variability with almost one-third of the athletes falling into the “poor sleeper” classification. These results are in agreement with the current literature quantifying sleep duration in (mainly non-athlete) adolescents. Additionally, to our knowledge, this is the first study to assess both current and ideal sleep behaviors in adolescent athletes from a variety of sports. Our results indicate there is a significant difference between these two measures, largely due to differences in desired bed and wake times.

In a review of global adolescent sleep patterns, the average total sleep time on school nights was less than 8 hours in 53% of the samples (n=18,157 across 14 included studies)^18^. Similarly, in the US-based NSF *Sleep in America* poll 62% of high-school aged adolescents (n=892) slept less than 8 hours on the average school night^19^. Several researchers have reported less than recommended sleep durations in adolescent athletes as well, albeit with a much smaller quantity of subjects than studies with non-athletes. Watson and Brickson^20^ reported 62% of the 60 female soccer players who self-reported sleep duration over a 1-year period slept on average less than 8 hours per night. Skein et al.^21^ assessed sleep habits during both a school/sports training period and a school break/ “off-season” sports training period, and reported an average nightly sleep duration of less than 8 hours (7:48 and 7:28 h:min, respectively) in 20 team-sport athletes. A similar sleep duration (7.7 h) has been reported in UK-based adolescent team-sport athletes^22^, while even less sleep has been reported in Swiss soccer players (7.0 h)^23^ and both Asian shooting and track and field sprinters (5.5 h)^6^. While sleep durations in adolescents do not seem to differ much between athletes and non-athletes, we also did not find any practical difference in sleep duration between the different sports included in our study or between male and female athletes. Additionally, we did not find any correlation between sleep duration and self-reported time spent training, meaning more training did not drive more sleep. In agreement with the current non-athlete literature^24^, we found a significant correlation between age and sleep duration; with sleep duration decreasing as age increases, likely a result of the later bedtime reported by older adolescents in our study. This is in line with biological factors thought to influence adolescent sleep patterns including a shift (delay) in the circadian rhythm as well as a slowing of sleep homeostatic pressure, increasing the length of time an individual can stay awake before feeling the need to sleep again^25^. This also supports the significant, positive correlation we found between age and *ideal* bedtime.

While quantity (duration) is a critical component of an athlete’s sleep behaviors, sleep quality also needs to be considered and evaluated, especially as it has previously been reported that despite similar sleep durations between (adult) athletes and non-athlete controls, sleep quality (including sleep latency and sleep efficiency) was significantly poorer in athletes^26^. In the current study we assessed sleep quality using the global PSQI score (0-21 scale with a score ≥ 5 indicative of poor sleep quality). Our results indicated, on average, most athletes have sufficient sleep quality, with no significant differences found between sports or sexes. That said, there was a large range in scores (0-15), with approximately 32% of the subjects scoring ≥ 5, highlighting the need to assess sleep individually when exploring potential areas of opportunity to improve performance and recovery in athletes. Although there is still quite limited research into the sleep quality of athletes, especially in adolescent athletes, our results appear to be in line or even more favorable than that of previously reported work.

A recent study of 98 elite athletes from a variety of sports (mean age 18.8 ± 3.0 y) reported a mean global PSQI score of 4.6 with approximately 41% of the athletes classified as “poor sleepers”^27^, while a systematic review of sleep quality in athletes reported a mean global PSQI score of 7^28^. It is hard to pin point what might be causing the difference, but it should be noted that all the studies in the review were adult athletes. Taking into account our findings that the global PSQI score was positively correlated with age, which has also previously been reported in a study of 309 adolescent athletes^7^, one may speculate age and/or maturation could play a role in the differences found.

Sleep latency, or the time in minutes to fall asleep, is a contributing factor to sleep quality and the global PSQI score, and was concomitantly also analyzed separately in our analysis. Previously, sleep latency has been shown to differ between athlete and non-athletes^26^ and thus may be a noteworthy sleep characteristic to assess in athletes. Mean sleep latency was 15 ± 11 minutes in the current study with no difference found between sports or sexes. This is in line with previous research with adolescent athletes (14 ± 10 and 11 ± 10 minutes for females and males, respectively)^29^ and Olympic athletes^26^, and may even be slightly more favorable than previous reports in other adolescent athletes^21^, as well as the large-scale NSF *Sleep in America* poll which reported an average sleep onset latency of almost 25 minutes^19^. However, similar to the global PSQI scores, there are individual differences that again may justify the importance of individually assessing sleep quality in athletes. Although not statistically significant, as a group baseball, football and lacrosse each had more than 20% of their athletes reporting a sleep onset latency of more than 30 minutes.

Despite a body of literature which suggests many adolescents are not meeting the recommended daily sleep durations as defined by professional organizations (i.e. CDC, ASSM, NSF, etc.), little attention has been paid to the *self-perceived ideal* sleep duration as defined by those actually in this population. Understanding how the target population perceives their need for sleep may have important implications, for example: in prescribing successful, behavior-changing interventions or educational programs to increase sleep duration, especially as the percentage of adolescents getting adequate sleep duration is likely declining^30^, despite the growing amount of literature on the importance of sleep. The athletes in our study reported an ideal sleep duration of almost 10 hours - the very highest end of the current sleep duration recommendations. With a gap of over two hours, this was significantly different from the amount of sleep the athletes were currently reporting, yet was consistent across all sports and sexes. Interestingly, the ideal sleep duration was the result of a combination of both a significantly earlier ideal bedtime and a significantly later ideal wake time. The later ideal wake time is in line with the circadian delay theory, however an earlier ideal bedtime may be considered somewhat surprising considering there is thought to be a biologically-controlled change in circadian timing during puberty which would drive a delay in sleep onset (bedtime)^25^.Ideal sleep time may be considerably more in the current study compared to findings from the NSF’s 2006 poll^19^ in which the adolescents reported 8.2 hours of sleep was needed, with only 38% of the adolescents reporting they needed 9 or more hours of sleep to feel their best. It is unknown what may be causing the difference in ideal sleep duration between the two groups of adolescents, but one possibility may be the impact of sports training, especially as there was a significant, positive correlation between training duration and ideal sleep duration in the current study. That said, whether sports training alone would add over 90 minutes to the ideal sleep duration is unknown as, to the best of our knowledge, there is no research on the influence of exercise on self-reported ideal sleep duration. Future research is warranted.

There are several limitations of the current study that should be acknowledged. First, all sleep variables, including sleep duration, were self-reported. Previous research has demonstrated a discrepancy between self-reported (subjective) and actigraphy measured (objective) sleep durations^31^, specifically demonstrating that adolescents *over*-estimate sleep duration. Therefore, it is quite possible the athletes in the current study are sleeping even less than they report, potentially making the gap between current sleep duration and both the clinical and self-reported ideal sleep durations even greater. In addition, while the current sleep habits of the athletes reported in this study are consistent with current literature^18-22^, it still should be noted that the athletes in the current study were living in a non-traditional (boarding school) environment and therefore may have different influencers (e.g. no on-site parental influence) and influences (e.g. the requirement to attend early morning practice, meals etc.) on their sleep behaviors. Finally, although there were no differences in any of the sleep variables between the sexes, females only made up approximately 20% of the athletes in the current study. While a better balance between the sexes would have been ideally obtained, this 20% mirrors the percentage of females in the overall boarding school demographic.

In conclusion, the present study suggests adolescent athletes, both male and female and from a variety of sports, are not meeting current sleep duration recommendations, with almost one-third of athletes also self-reporting poor sleep quality as determined by global PSQI scores. Noteworthy, while current sleep durations are missing even the lowest end of the recommendations, self-reported ideal sleep duration is at the very top-end of the 8-10 hour recommendations. Future research should focus on the cause (and effect) of this discrepancy in an attempt to set forth relevant and realistic interventions to increase sleep duration in this population and to, ultimately, impact performance, health and wellbeing.
